# A diagnostic challenge: primary omental torsion and literature review - a case report

**DOI:** 10.1186/1749-7922-4-40

**Published:** 2009-11-18

**Authors:** Nina Breunung, Paul Strauss

**Affiliations:** 1 General Surgery, Darent Valley Hospital, Dartford & Gravesham NHS Trust, Dartford, Kent, UK

## Abstract

A case report of omental torsion, which is a rare differential diagnosis of the acute abdomen. Intraoperative diagnosis and treatment by resection are the current management of choice, however with increasing use of pre-operative imaging this may need to be reconsidered.

## Background

The four layered fatty sheet of peritoneum is known as omentum and suspends from the greater gastric curvature to surrounding organs with attachments to the diaphragm [1]. Omental torsion is caused by twisting of sections of the omentum along its long axis resulting in vascular compromise. First described by Eitel in 1899 it is a rare cause of the acute surgical abdomen [2,3]. Fewer than 250 cases have been described in the literature so far. Omental torsion is rarely diagnosed preoperatively and may lead to spontaneous clinical deterioration of the patient [2,4]. Laparoscopy is the current choice for diagnosis and management [5].

## Case History

A 44 year old female patient presented to the Emergency Department complaining of generalised abdominal pain for three days, localising to the right iliac fossa. Accompanying symptoms were nausea and constipation, but bowels had opened on day of presentation. No urinary symptoms, past medical history of note or regular medication were present.

On examination the patient was haemodynamically stable and apyrexial. The abdomen was soft, not distended, with localised tenderness in the right iliac fossa without peritonitis. Apart from a mild leukocytosis (11.2 × 10^9^/L), the blood count and serum biochemistry were normal on first presentation.

She was initially discharged home, but returned the following day with unresolving symptoms and was referred to the surgical team.

Abdominal ultrasound was normal and no appendix mass identified. After two days of observation and non resolving symptoms the patient underwent diagnostic laparoscopy, with a suspicion of appendicitis.

**Figure 1:**
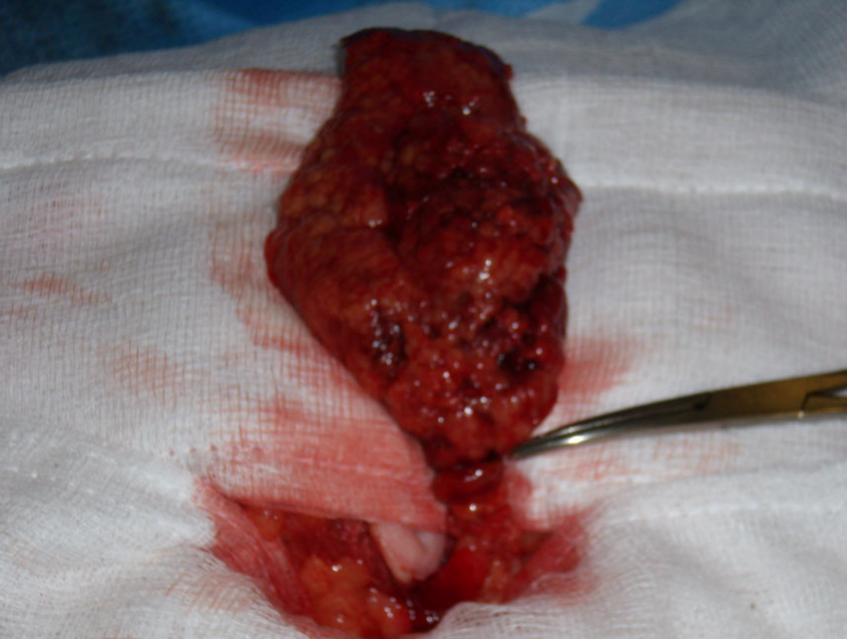
**Operative picture demonstrating torted omentum section with three twists**.

On laparoscopy a small amount of blood stained fluid and an inflammatory mass consisting of a section of infarcted omentum and adherent thickened small bowel were identified. Appendix, gallbladder and pelvis showed no abnormality. The procedure was extended to a mini-laparotomy. The inflammatory mass was dissected and identified as an omental torsion with three twists (Figure 1). The small bowel was normal and intact. The infarcted omentum was resected.

**Figure 2:**
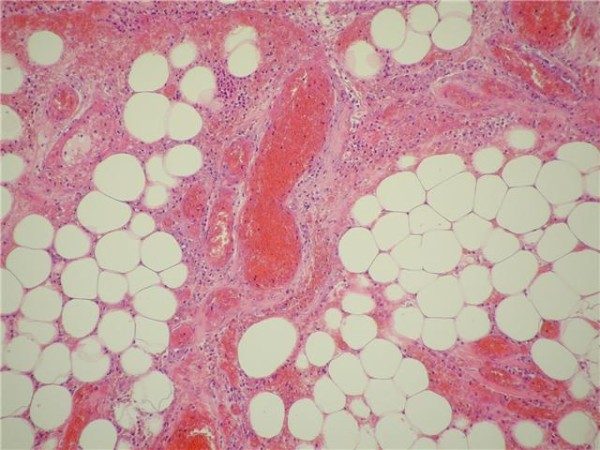
**Histology displaying omental torsion characterised by congested vessels, inflammation, necrosis (ischaemic and fat) and fibrinoid exudates**.

**Figure 3:**
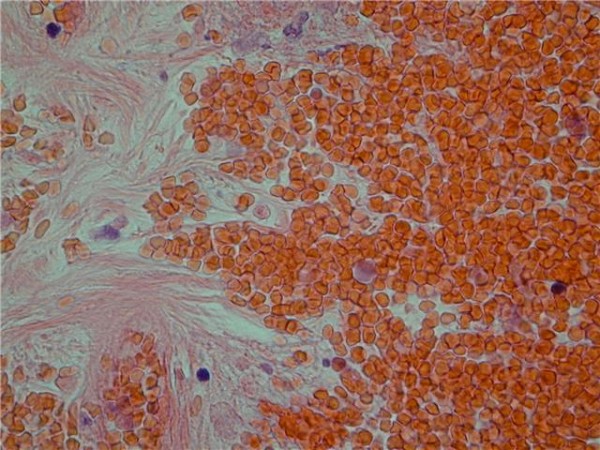
**Histology displaying omental torsion characterised by congested vessels, inflammation, necrosis (ischaemic and fat) and fibrinoid exudates**.

Post-operative recovery was without complications and the patient was discharged home two days after surgery. The histology findings confirmed omental torsion characterised by congested vessels, inflammation, necrosis (ischaemic and fat) and fibrinoid exudates (Figures 2 &3).

## Discussion

Omental torsion is a rare cause of abdominal pain presenting mainly in the 3^rd ^to 5^th ^decade of life with a slight male predominance (3:2) [5,6]. The omentum twists around its long axis, clockwise at a pivotal point. Consequently vascularity is compromised, resulting in haemorrhagic extravasation, serosanguinous fluid production, necrosis and adhesion formation.

Omental torsion may be primary or secondary. One third of cases are a result of primary torsion, which is unipolar with no underlying pathology or distal fixation [5-7]. In primary torsion the volvulus occurs more commonly around the right distal epiploic artery due to greater size and mobility of the omentum in this region [1,2]. Factors such as anatomical variations in the omentum and actions that displace the omentum such as trauma, exercise or hyperpersitalsis predispose to torsion. Obesity has also been implemented as a risk factor [1,8]. Secondary torsion is more common and a result of underlying abdominal pathology (e.g. cysts, adhesions, hernial sacs) resulting in a distal fixation point (bipolar torsion) [2,7]. In some cases the omentum may infarct without torsion, which is known as primary idiopathic segmental infarction [6].

Patient with omental torsion present with constant, non-radiating pain of increasing severity, nausea and vomiting. Clinically 50% of patients have a low grade fever and leukocytosis [4,5]. These findings are non specific, making pre-operative diagnosis of omental torsion a challenge. The majority of cases present with a single episode of abdominal pain but recurrent pain may suggest intermittent torsions [4,9]. On examination 50% of patients present with an abdominal mass and localised peritonitis [5,7]. Common differential diagnosis include appendicitis, cholecystitis or twisted ovarian cyst [2]. In general patients with omental torsion are less systemically unwell compared to acute appendicitis and the disease process extends over a longer period of time [6].

On laboratory findings a moderate leukocytosis is present in 50% of cases [2]. Imaging investigations such as Ultrasonography and Computed Tomography (CT) have been suggested in the literature [10]. On Sonography a complex mass consisting of hypoechoic and solid zones may be identified, but this imaging technique is operator dependent with limited sensitivity due to overlying bowel gas. On CT, omental torsion is characterised by diffuse streaking in a whirling pattern of fibrous and fatty folds [2,10]. With increased use of CT, pre-operative diagnosis of omental torsion may increase in frequency of preoperative diagnosis and lead to conservative management in patients without complications [8,10-12].

The current investigation tool and therapeutic management of choice is laparoscopy proceeding to laparotomy, identifying and removing the infarcted section of omentum. Normal appendix, gallbladder and pelvic cavity make the diagnosis of omental torsion likely. Free serosanguineous fluid as a result of haemorrhagic extravasion is a characteristic finding in the peritoneal cavity. In the literature the treatment of choice included additional appendicectomy to prevent future diagnostic problems. Successful conservative management has also been reported [5,13].

Histology findings of haemorrhagic infarction and fat necrosis confirm the diagnosis with the presence of fibrosis indicative of a longer disease process [4].

The prognosis for primary omental torsion is good with fast post operative recovery and minimal morbidity. The natural disease progress if left untreated will result in fibrosis, necrosis and occasional autoamputation and clinical improvement [7,14]. Prognosis in secondary torsion depends in the underlying pathology.

Left sided omental torsion may be commonly misdiagnosed as diverticulutis and managed conservatively, resulting in less common diagnosis [7].

## Conclusion

Omental torsion presents with non specific symptoms of an acute abdomen and is mainly diagnosed intraoperatively during diagnostic laparoscopy. Awareness of omental torsion as a differential diagnosis in the acute abdomen and careful inspection of omentum in a "negative laparoscopy" are recommended for appropriate management of the surgical patient [4]. However cases without complications, may be managed conservatively in future [10].

## Competing interests

The authors declare that they have no competing interests.

## Authors' contributions

NB performed the literature review and drafted the paper. PS reviewed the manuscript and provided the figure. The manuscript was read and approved by all authors.

## Consent

Written informed consent was obtained from the patient for publication of this case report.
